# Perceived Risk in the Population Living near the Turin Incinerator: Comparison between before and at Three Years of Operation

**DOI:** 10.3390/ijerph18179003

**Published:** 2021-08-26

**Authors:** Antonella Bena, Martina Gandini, Laura Crosetto, Cristiana Ivaldi, Enrico Procopio, Giuseppe Salamina, Manuela Orengia, Elena Farina

**Affiliations:** 1 Regional Epidemiology Unit, ASL TO3, Piedmont Region, Via Sabaudia 164, 10095 Grugliasco, Italy; antonella.bena@epi.piemonte.it (A.B.); elena.farina@epi.piemonte.it (E.F.); 2 Environmental Epidemiological Unit, Regional Environmental Protection Agency, Piedmont Region, Via Pio VII 9, 10135 Turin, Italy; laura.crosetto@arpa.piemonte.it (L.C.); cristiana.ivaldi@arpa.piemonte.it (C.I.); manuela.orengia@arpa.piemonte.it (M.O.); 3 Department of Prevention, ASL TO3, Piazza San Francesco 4, 10059 Susa, Italy; eprocopio@aslto3.piemonte.it; 4 Department of Prevention, ASL TO1, Via Della Consolata 10, 10122 Turin, Italy; giuseppe.salamina@aslcittaditorino.it

**Keywords:** perceived risk, incinerator, pre–post analysis, human biomonitoring survey

## Abstract

When the Turin incinerator went into operation in 2013, it was accompanied by surveillance of health effects that included a human biomonitoring survey of 394 residents. They responded to items investigating their awareness of environmental and health issues and perception of environmental health risks. In this study, we compared the questionnaire responses before plant startup and at 3 years of operation. To accomplish this, we investigated changes in perceived risk and evaluated the efficacy of communication strategies. A total of 344 participants equally distributed in an exposed and an unexposed group responded to the follow-up questionnaire. Survey items investigated the perception of a relationship between illness and exposure to environmental pollution, feeling at risk of developing an illness, and concern about natural and anthropogenic hazards. The proportion of ‘certain’ and ‘very probable’ responses was compared to the total using the difference-in-differences method. Analyses showed an overall decrease in the differences between the two groups, which suggests that the communication actions undertaken for the exposed group were effective. Future communication plans should also include initiatives targeting the unexposed group.

## 1. Introduction

Risk perception of the health impact of waste incinerator emissions is generally very high [1,2], often assuming greater dimension in relation to the risks involved [3,4]. Risk perception of environmental factors can be interpreted as a combination of objective (e.g., levels of real exposure to a hazard) and subjective factors (e.g., assessment arising from education, culture, values, personal beliefs, perception of reality) [5]. Risk perception differs by cultural background [6], social, political, and decision-making dynamics [4,7], and trust in the company that operates the plant and the public agencies responsible for its surveillance [8,9].

With heightened public sensitivity towards environmental problems, citizens increasingly demand that agencies provide an account of supervision, promote independent research, present data, and build their own information tools [10]. Effective risk communication can play a pivotal role in the management of potential conflicts between institutions and population, especially where public concern is high, but the potential risk is quite low [11,12]. Incinerator hazards is a deeply felt topic since waste combustion processes might generate emissions of organic and inorganic micropollutants, including polychlorinated dibenzodioxins (PCDDs) and dibenzofurans (PCDFs) (together also known as ‘dioxins’), polychlorinated biphenyls (PCBs), polycyclic aromatic hydrocarbons (PAHs), and metals. The European Directive 2000/76/CE regulated the requirements for waste incineration plants as well as the emission limits for certain pollutants.

Clear and accurate communication is often hindered by easy accessibility to multiple sources of variable quality and conflict-seeking media. To provide balanced counterarguments, risk communication needs to combine technical expertise with community values and preferences in a rational decision-making process [13] guided by conventional strategies (meetings, newsletters, websites) and modern communication tools (risk perception surveys, structured opportunities for stakeholder engagement, discussion with decision-makers). Accordingly, the questionnaire for the human biomonitoring (HBM) survey in the SPoTT (Italian acronym for Population Health Surveillance in the Turin Incinerator Area) study [14] incorporated a section on risk perception.

The responses collected before the waste-to-energy (WTE) plant went into operation (T0, 2013) were a key factor in defining suitable communication strategies both for residents living near the plant and an unexposed group residing about 3–7 km from WTE, and not interested by its emissions, according to fallout maps [15]. Items on risk perception were repeated in the questionnaire administered in the second follow-up of the HBM survey (T1, 2014; T2, 2016). The difference in risk perception after the HBM results and the plant emission monitoring data was analyzed to determine the effectiveness of the communication strategy. This was crucial to understanding the attitude of the local citizens, given the habituation of living with risk in some cases [3] and growing concern in others about health effects and issues related to waste transport [16].

In this paper, we evaluated the effectiveness of risk communication strategies by comparing the responses to a risk perception questionnaire, which residents completed before and at 3 years into the operation of the incinerator. This study is a part of a wider surveillance system on local residents, which included HBM, evaluation of adverse short-term health effects in terms of emergency room accesses and hospital admissions, together with ongoing surveillance on long-term effects. Moreover, there is a daily monitoring system about WTE emissions and their fallout. The risk perception survey within the SPoTT program provides the opportunity to check whether the communication strategy with citizens about this surveillance system and new issues in the scientific literature were effective or not. To our best knowledge, this is the first study to compare the perceived risk of local residents before and after a WTE plant went into operation.

## 2. Materials and Methods

### 2.1. Characteristics of Incinerator Area

WTE is located in an industrial area of around 100,000 m^2^ in which about 100,000 people live. This is an area already subject to environmental, industrial pollutants, and vehicular traffic. WTE burns urban solid waste, as well as special waste that can be assimilated to the first category, converting the heat produced by combustion into thermal and electric energy. Three identical independent lines can burn a total of 421,000 tons of waste per year. The testing activity on lines 1 and 2 began in April 2013 but was stopped from June to August 2013 to allow the execution of the biomonitoring program. WTE became fully operational in July 2014.

**Figure 1:**
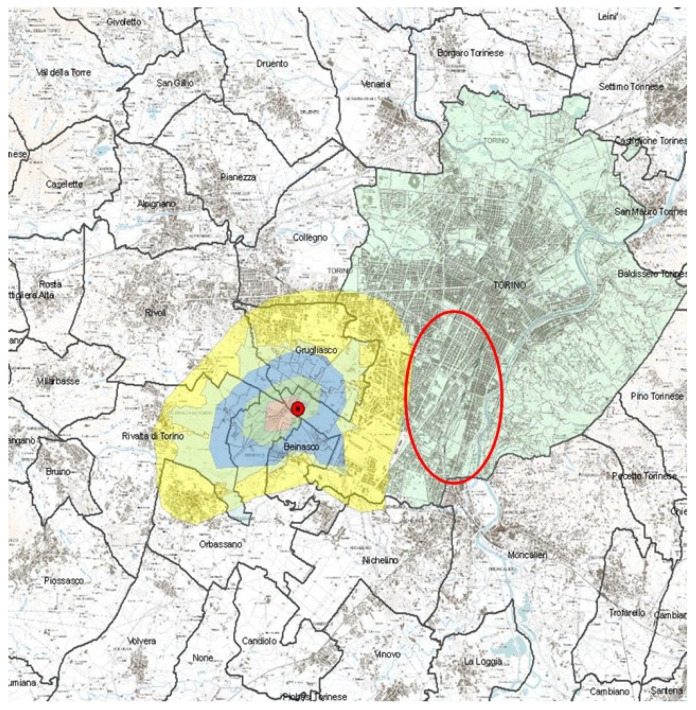
Fall-out map. Red dot is the WTE plant location; green area is the municipality of Turin. Blue area corresponds metals depositions >0.014 mg/m^2^/year (exposed group). Red ellipse corresponds to the area of Turin municipality where control group is sampled (unexposed group).

To determine the WTE fallout, a CALPUFF Lagrangian particle software model (v 6.42, Exponent Inc., Menlo Park, CA, USA), which included the CALMET (v 5.8, Exponent Inc., USA) diagnostic wind model and the CALPOST post-processor (v 6.291, Exponent Inc., USA), was used, giving estimates of metal deposition for each address of the study area (Figure 1). Forecasting model for PCB and PCDD/F is very similar, so metal deposition maps were chosen to define the two areas.

### 2.2. Questionnaire

Before the incinerator became fully operational (T0), the SPoTT HBM questionnaire was administered by trained personnel to 394 local residents (age range, 35–69 years) living for at least 5 years in two areas: 198 in an area with metals depositions >0.014 mg/m^2^/year (exposed group); 196 in an area with metals depositions <0.007 mg/m^2^/year (unexposed group). The study participants were sampled randomly from the municipal registry, stratified by sex and 5-year age groups [14]. At the second survey (T2), after 3 years of WTE plant operation, the questionnaire was readministered to 344 out of the 394 residents sampled at T0, equally distributed between exposed and unexposed (172 subjects among exposed and 172 among exposed). A total of 50 subjects were lost to follow-up due to changes in residence address (17 subjects), deaths (2), or unwillingness to further participation (31).

The questionnaire section on risk perception included three sets of items, investigating perception of environmental hazards (more detail available in [15]):

Q1—Which of the following do you think are caused by environmental pollution?
Q2—Do you think you risk developing these diseases?
Q3—Which of these events concern or disturb you most?

Questions Q1 and Q2 investigated diseases such as allergies, acute and chronic respiratory diseases, temporary illness, liver damage, cancer, leukemia, congenital defects. For question Q3, we investigated natural environmental calamities (severe weather events, floods, earthquakes) and anthropogenic hazards (transportation-hazardous materials, nuclear events, hazardous industries, air pollution, water pollution, waste management, noise, fires). The HBM questionnaire first part provided sociodemographic data and the health status (e.g., sex, age, marital status, geographical area of birth, educational level, occupation, smoking and alcohol consumption, having had children, self-perceived health, etc.). Upon enrolment, study participants received an informed consent form explaining the study objectives and procedures and treatment of personal data. Participants were informed about the possibility of withdrawing from the study at any time.

### 2.3. Statistical Analysis

Each item was graded dichotomously: 0 = not very, not at all and 1 = extremely, very; 0 = quite probable, not very probable, and 1 = certain, very probable. The ‘do not know’ responses were not considered, and the focus of our analyses is on worried people. Two further indicators were added: one to summarize worries about anthropogenic hazards (1 = worried about at least five of the corresponding items and 0 = otherwise), and another one to sum up natural hazards (1 = worried about at least two of the corresponding items and 0 = otherwise).

The absolute frequency and the percentage for each category were calculated on the total sample of 344 respondents. T0 lost to follow-up participants were excluded from the main analyses. A sensitivity analysis has been completed, including their contribution that is only for T0; results of this analysis are not reported since their inclusion do not show relevant modification when interpreting the results.

Difference-in-differences (DID) analyses [17,18] were performed to determine whether differences in risk perception changed over time (from T0 to T2) in a differential way between the exposed and the unexposed group. In its basic formulation, DID is based on two groups and two periods. In the first period, none of the groups is exposed to treatment, while in the second period, only one of the two groups is exposed to treatment. In our situation, the ‘treatment’ is exposure to WTE. It is a technique widely used for impact evaluation [19,20]. The DID model is illustrated below:

$$Y=\beta_{0}+\beta_{1}T+\beta_{2}P+\beta_{3}\left(T\times P\right)+\beta_{4}S_{i}+\varepsilon$$

where *T* is a dichotomous variable indicating group (*T* = 0 unexposed and *T* = 1 exposed); *P* is a dichotomous variable indicating time period (*P* = 0 T0 and *P* = 1 T2); (*T* × *P*) is the interaction between the intervention and the time period; *S_i_* is a vector of control variables. The coefficient which provides the DID estimate is *β*_3_. It quantifies the difference in trend between the two time periods. Each item of the questionnaire was tested with a different equation, and each coefficient has its own *p*-value, which suggests the statistical significance of the DID estimate. The use of the DID methodology is particularly useful when the trend is the same in the two groups, allowing evaluation of whether the difference is significant or not.

Control variables to be entered in the DID model were chosen from those recorded in the first part of the questionnaire: sex, age (35–45, 46–55; 55+), geographical area of birth (north, center-south; abroad), level of education (primary or secondary school, high school, college), marital status (single or widow(er); married/cohabiting, separated/divorced), presence of children (yes, no), self-perceived health (good health; poor health). Since individuals have been enrolled by municipal registry, individuals have been sampled by sex and age. Chi-square tests have been performed to check whether there may be differences between groups according to the other variables. Although recorded, employment status was not investigated since 42 subjects in the exposed group and 55 subjects in the unexposed group did not declare their professional qualification. Potential control variables which change significantly from T0 to T2 were entered in the DID model, while the other variables are not expected to have an influence when comparing differences.

Statistical significance was set at *p* < 0.05. Analyses were performed using STATA 13 software (StataCorp. 2013. Stata Statistical Software: Release 13. College Station, TX, USA: StataCorp LP).

The use of personal data was carried out in compliance with current Italian legislation. All information collected and recorded on electronic devices was anonymized by an identifying code number. The archive containing the names with the code number was held separately.

## 3. Results

Despite some loss to follow-up, the distribution by sex and age (mean age = 55.2 years in both groups) for the two groups remained substantially unchanged (Table 1). As in the T0 survey, there were more graduates in the unexposed group, while the number of married/cohabiting and those who had children was higher in the exposed one. The only variable which changed significantly from T0 to T2 was the educational level (*p* = 0.0008, chi-square test), therefore being the only control variable entered in the DID model.

**Figure 2:**
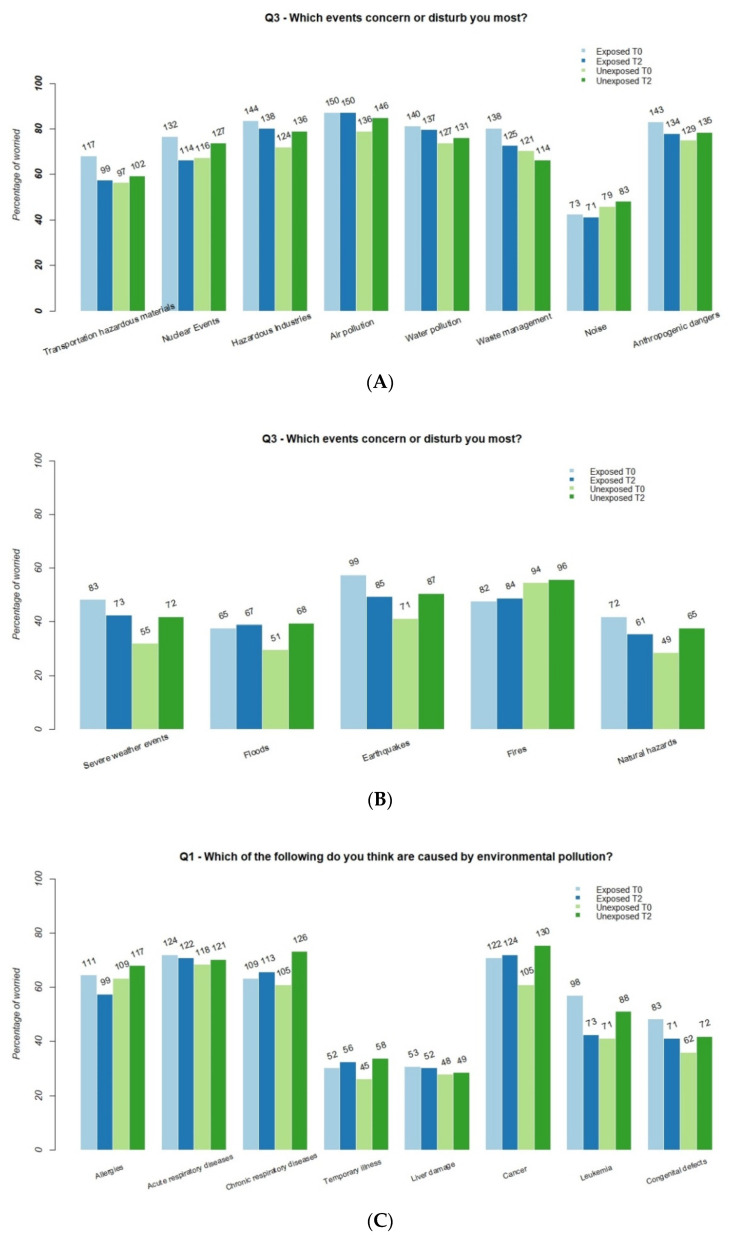

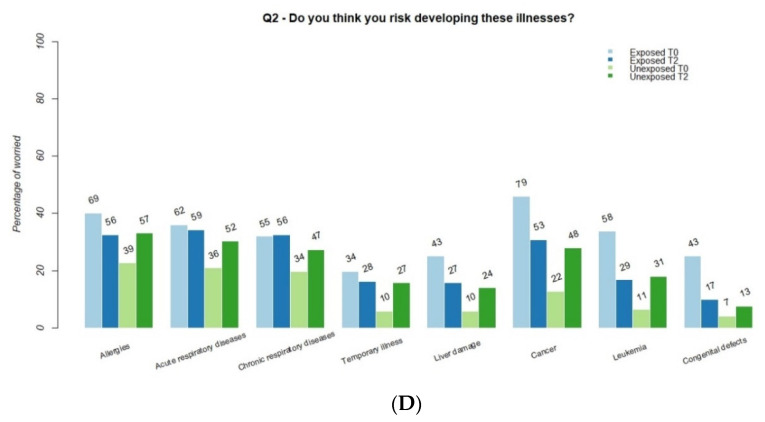
(Panels **A**–**D**) Change in percentage of concern for the two groups (extremely, very; or certain, very probable) from T0 (before WTE start-up) to T2 (after three years of functioning).

After 3 years of functioning, concern about waste management was slightly lower for both groups (Figure 2A), albeit higher for the exposed group. Concern about anthropogenic hazard was lower for the exposed and slightly higher for the unexposed group (Figure 2A) for all the items, with the exception of waste management. Overall concern about natural hazards was lower for the exposed and higher for the unexposed group (Figure 2B). We observed a decrease in the level of concern among the exposed and a general increase among the unexposed for the responses to the items ‘Which of the following do you think are caused by environmental pollution?’ (Q1) and ‘Do you think you risk developing these illnesses?’ (Q2) (Figure 2C,D).

From Figure 2A–D it becomes clear that, for some of the investigated issues, there is a marked difference in perceived risk over time among the two groups. The DID model tests this assumption for each item. For item Q1, the changes were only statistically significant for leukemia caused by air pollution (DID −0.244, *p* = 0.001). For item Q2, the concern about being at risk of developing an allergy (DID −0.181, *p* = 0.011), temporary illness (DID −0.133, *p* = 0.013), liver damage (DID −0.172, *p* = 0.001), various form of cancer (DID −0.300, *p* < 0.001), leukemia (DID −0.282, *p* < 0.001), or congenital defects (DID −0.184, *p* < 0.001) was higher for the unexposed and lower for the exposed group (Table 2, Q1).

For the items ‘Which events concern or disturb you most?’ (Q3), we observed a similar decline. The differences in variation were significant for severe weather events (DID −0.151, *p* = 0.040), earthquakes (DID −0.168, *p* = 0.025), and nuclear events (DID −0.164, *p* = 0.017) (Table 2, Q3). We noted a decrease in waste management concern for both groups: a decrease from 80% to 73% for the exposed and from 70% to 66% for the unexposed group, but not statistically significant.

## 4. Discussion

The responses to the survey conducted before the WTE plant went into operation (T0, 2013) showed greater concern among residents living closer to the incinerator than those living far from it [15]. This was expected and consistent with previous studies that reported an inversely proportional relationship between the increase in risk perception and the increase in distance from an incinerator, with the willingness to move in a short time far from the incinerator when construction of a new one is planned [3,9,21]. The topic analyzed in this paper is quite different from other studies due to the nature of the area involved. Particularly, before WTE startup, there was great concern about health risks, particularly for cancer, leukemia, and congenital defects, which are rare events, but there was evidence in the literature as adverse health effects associated with old plants emissions [22,23]. It is worth noting that both groups live in an urban area with a similar urban background of traffic and industrial emissions. The SPoTT working group carried out communication actions to improve knowledge and involvement of the local community on pollution and health issues. Over the next 3 years, SPoTT developed communication tools in the periodically updated communication plan [24]. The HBM study protocol, the results reports, and other documentation on the SPoTT program are available in a dedicated web area (www.dors.it/spott (accessed on 15 July 2021)).

Collective and individual meetings were held to communicate the results to the participants in the biomonitoring survey. The communication plan was coordinated yet independent from the communication actions of the local agencies and the WTE plant owner. Before the WTE plant went into operation, a monitoring committee was formed and composed of local civil agencies and health and environmental technicians in addition to the WTE company, which participated by invitation. It held public meetings to share with the public the progress of the SPoTT program, with particular attention to the citizens living near the plant. Press releases were prepared to present the results. With the SPoTT program, we had the opportunity to have a pre–post comparison for HBM and questionnaire associated with it, an issue not investigated in other studies, where the long-term existence of a specific pollution source does not always permit having a temporal comparison.

When the WTE plant started up, the two groups differed widely in their beliefs about the link between atmospheric pollution and diseases, especially cancer, leukemia, and congenital malformations. Three years later, their beliefs were more aligned with each other. All the DID were negative, although only the DID for leukemia was statistically significant (Table 2, Q1). At T0, the difference was more pronounced in concern about developing an illness because of environmental pollution (Figure 2B). The between-group difference diminished over time, and the reduction was statistically significant for all of the illnesses listed in the questionnaire, with the exception of respiratory diseases (both acute and chronic) (Table 2, Q2). This can be partially explained mainly by their association with overall sources of environmental pollution, not strictly related to incinerators or other polluted sites.

Both groups retained greater concern about anthropogenic hazards than natural disasters; this observation is shared by previous studies on the Italian population in areas affected by anthropogenic pollution and industrial sites [6,25,26]. The differences between the two groups decreased with time (Table 2, Q3); for example, the between-group difference in concern about waste management decreased. Though not statistically significant, the decrease concerned both groups (Figure 2D). In this study, we did not investigate perceived risk associated with food as in other studies [26], since it is mainly an urban area and only a small amount of the sample (about 15%) consumed local products and only occasionally.

A plausible explanation for these changes is the pre-existing risk perception, as revealed by the responses to the questionnaire before WTE startup, in combination with the knowledge acquired later and level of involvement in risk communication activities [7]. People often find it difficult to access, understand, and utilize health information: 12% of the European population has a low health literacy level and understanding health information is problematic for 35% of the population. Italy ranks at the lower end of the classification [27,28].

Planning of risk communication was completed at the time the WTE plant went into operation and is still ongoing. Such continuity may have played a key role in reducing the difference in risk perception between the two groups. The plan defined a set of communication rules, identified the population segments to be targeted, defined the communication strategies appropriate for each group, and set realistic deadlines for the project objectives. The plan was transparent; it was presented and discussed with all stakeholders. The working group met the deadlines and analyzed and explained the reasons for delays in the project. The communication strategy was not limited to build trust but rather clearly defined the procedures and standards in a comprehensible way and acceptable to the stakeholders [29].

These efforts seemed to have an impact on the perceived risk of the group residing near the WTE: the decrease in the between-group difference is linked to the decrease in concern of overexposure by the one group and an increase in awareness by the other (Figure 2). This finding should be interpreted within the environmental context in which the plant is located: an area with heavy air pollution. During the years in which the study was conducted, higher PM10 levels were recorded for the area where the unexposed group resided [30], and this may explain the increase in the perception of being at risk of developing a set of illnesses (Figure 2D). In addition, the HBM study reported an increase in metals from motor vehicle and industrial emissions [31]. Given these circumstances, the SPoTT working group has decided to involve the unexposed group in future communication initiatives.

The major strength of the present study is the random sampling according to municipal registries: the two groups are representative of the local population and similar to one another [14]. Moreover, the two groups are similar in demographic characteristics that could influence risk perception. To our best knowledge, this is the first longitudinal study comparing risk perception by local residents before and after a WTE plant went into operation. The questionnaire will be administered again in a survey scheduled for 2022. Caution should be taken in the interpretation of the results, with the pandemic as a possible confounding factor in the trend’s explanation.

## 5. Conclusions

The aim of this paper was to evaluate the effectiveness of risk communication strategies on environmental hazards. This has been completed by submitting a questionnaire to local residents involved in HBM before WTE startup and after 3 years of operation. The survey has been administered to an exposed group and an unexposed one, sharing similar environmental exposures and socio-demographical characteristics. Analyses have been performed using DID methodology to test differences in perceived risk in the two groups. The decrease in the between-group differences suggests that the risk communication actions were effective. During these years, residents have been informed of the real and possible damages, and communication with citizens has been completed periodically, sharing results of human biomonitoring and of the other health surveillance activities included in the SPoTT program (potential short-term adverse health effects and surveillance on workers). Communication plans should include initiatives targeting groups that are exposed or not to air pollution. The SPoTT program will continue for other 4 years of health surveillance, with another follow-up of the HBM activity. Further researches will be completed to check changes in perceived risks concerning environmental issues.

## Figures and Tables

**Table 1:** Sociodemographic characteristics at T2 (N = 344).

Variable	Unexposed		Exposed		Total	
	N	%	N	%	N	%
Sex						
Males	89	51.7	85	49.4	174	50.6
Females	83	48.3	87	50.6	170	49.4
Geographical area of birth						
North	107	62.2	117	68.0	224	65.1
Center-South and Islands	53	30.8	49	28.5	102	29.7
Abroad	12	7.0	6	3.5	18	5.2
Marital status						
Married/cohabiting	132	77.2	143	83.1	275	80.2
Separated/divorced	9	5.3	13	7.6	22	6.4
Single/widow(er)	30	17.5	16	9.3	46	13.4
Had children (yes)	131	76.2	143	83.1	274	79.7
Level of education						
College	44	25.7	19	11.0	63	18.4
High school	81	47.4	85	49.4	166	48.4
Middle/primary school	46	26.9	68	39.5	114	33.2
Self-perceived health						
Good state of health	132	77.2	143	83.1	275	80.2
Poor state of health	9	5.3	13	7.6	22	6.4

**Table 2:** Difference-in-differences (DID) results controlled for educational level. Absolute values and percentage of worried people are reported for each item of the questionnaire.

**Q1—Which of the Following Do You Think are Caused by Environmental Pollution?**	**Exposed** **T0 ^1^**	**Exposed** **T2**	**Unexposed** **T0**	**Unexposed** **T2**	**DID Estimate**	***p*-Value**
Allergies	111 (64.5%)	99 (57.6%)	109 (63.4%)	117 (68.0%)	−0.119	0.107
Acute respiratory diseases	124 (72.1%)	122 (70.9%)	118 (68.6%)	121 (70.3%)	−0.030	0.668
Chronic respiratory diseases	109 (63.4%)	113 (65.7%)	105 (61.0%)	126 (73.2%)	−0.102	0.156
Temporary illness	52 (30.2%)	56 (32.6%)	45 (26.2%)	58 (33.7%)	−0.052	0.464
Liver damage	53 (30.8%)	52 (30.2%)	48 (27.9%)	49 (28.5%)	−0.011	0.873
Cancer	122 (70.9%)	124 (72.1%)	105 (61.0%)	130 (75.6%)	−0.134	0.055
Leukemia	98 (57.0%)	73 (42.4%)	71 (41.3%)	88 (51.2%)	−0.244	0.001
Congenial defects	83 (48.3%)	71 (41.3%)	62 (36.0%)	72 (41.9%)	−0.128	0.088
**Q2—Do You Think You Risk Developing These Illnesses?**	**Exposed** **T0**	**Exposed** **T2**	**Unexposed** **T0**	**Unexposed** **T2**	**DID Estimate**	***p*-Value**
Allergies	69 (40.1%)	56 (32.6%)	39 (22.7%)	57 (33.1%)	−0.181	0.011
Acute respiratory diseases	62 (36.0%)	59 (34.3%)	36 (20.9%)	52 (30.2%)	−0.108	0.121
Chronic respiratory diseases	55 (32.0%)	56 (32.6%)	34 (19.8%)	47 (27.3%)	−0.068	0.321
Temporary illness	34 (19.8%)	28 (16.3%)	10 (5.8%)	27 (15.7%)	−0.133	0.013
Liver damage	43 (25.0%)	27 (15.7%)	10 (5.8%)	24 (13.9%)	−0.172	0.001
Cancer	79 (45.9%)	53 (30.8%)	22 (12.8%)	48 (27.9%)	−0.300	0.000
Leukemia	58 (33.7%)	29 (16.9%)	11 (6.4%)	31 (18.0%)	−0.282	0.000
Congenial defects	43 (25.0%)	17 (9.9%)	7 (4.1%)	13 (7.6%)	−0.184	0.000
**Q3—Which Events Concern or Disturb You Most?**	**Exposed** **T0**	**Exposed** **T2**	**Unexposed** **T0**	**Unexposed** **T2**	**DID Estimate**	***p*-Value**
Sever weather events	83 (48.3%)	73 (42.4%)	55 (32.0%)	72 (41.9%)	−0.151	0.040
Floods	65 (37.8%)	67 (39.0%)	51 (29.7%)	68 (39.5%)	−0.081	0.262
Earthquakes	99 (57.6%)	85 (49.4%)	71 (41.3%)	87 (50.6%)	−0.168	0.025
Noise	73 (42.4%)	71 (41.3%)	79 (45.9%)	83 (48.3%)	−0.035	0.645
Fires	82 (47.7%)	84 (48.8%)	94 (54.7%)	96 (55.8%)	0.002	0.976
Natural hazards	72 (41.8%)	61 (35.5%)	49 (28.5%)	65 (37.8%)	−0.155	0.032
Transportation of hazardous materials	117 (68.0%)	99 (57.6%)	97 (56.4%)	102 (59.3%)	−0.131	0.079
Nuclear events	132 (76.7%)	114 (66.3%)	116 (67.4%)	127 (73.8%)	−0.164	0.017
Hazardous industries	144 (83.7%)	138 (80.2%)	124 (72.1%)	136 (79.1%)	−0.104	0.094
Air pollution	150 (87.2%)	150 (87.2%)	136 (79.1%)	146 (84.9%)	−0.059	0.283
Water pollution	140 (81.4%)	137 (79.7%)	127 (73.8%)	131 (76.2%)	−0.039	0.535
Waste management	138 (80.2%)	125 (72.7%)	121 (70.3%)	114 (66.3%)	−0.035	0.608
Anthropogenic dangers	143 (83.1%)	134 (77.9%)	129 (75.0%)	135 (78.5%)	−0.088	0.129

^1^ T0: before WTE start-up, T2: after three years of WTE functioning.

## Data Availability

The data presented in this study are available on request from the corresponding author. The data are not publicly available due to privacy reasons.
